# Supportive Care Needs in Glioma Patients and Their Caregivers in Clinical Practice: Results of a Multicenter Cross-Sectional Study

**DOI:** 10.3389/fneur.2018.00763

**Published:** 2018-09-11

**Authors:** Mirjam Renovanz, Dorothea Maurer, Heike Lahr, Elke Weimann, Monika Deininger, Christian Rainer Wirtz, Florian Ringel, Susanne Singer, Jan Coburger

**Affiliations:** ^1^Department of Neurosurgery, University Medical Center, Johannes Gutenberg University Mainz, Mainz, Germany; ^2^Department of Neurology, Klinikum Ludwigsburg, Ludwigsburg, Germany; ^3^Department of Neurosurgery, University Medical Center Ulm, Günzburg, Germany; ^4^Division of Epidemiology and Health Services Research, Institute of Medical Biostatistics, Epidemiology and Informatics, University Medical Center, Johannes Gutenberg University Mainz, Mainz, Germany

**Keywords:** supportive care needs, palliative care, glioma, brain tumor, self-assessment

## Abstract

**Objective:** Supportive care needs in glioma patients often remain unrecognized, and optimization in assessment is required. First, we aimed at assessing the support needed using a simple structured questionnaire. Second, we investigated the psychosocial burden and support requested from caregivers.

**Methods:** Patients were assessed at three centers during their outpatient visits. They completed the Distress Thermometer (DT; score ≥ 6 indicated significant burden in brain tumor patients), the European Organization for Research and Treatment of Cancer Quality of Life Questionnaire (EORTC QLQ)-C30+BN20, and the Patients' Perspective Questionnaire (PPQ) that assessed psychosocial distress as well as support requested and received by patients for specific domains (e.g., family, doctor, and mobile care). In each subgroup, patients' caregivers were assessed simultaneously by a questionnaire developed for the study. Multivariate backward logistic regressions were performed for investigating predictors of patients' request for support.

**Results:** Assessments were conducted for 232 patients. Most patients (82%) had a high-grade glioma and a mean age of 52 years (range 20–87). The male to female ratio was 1.25:1. According to the PPQ results, 38% (87) of the patients felt depressed; 44% (103), anxious; and 39% (91), tense/nervous. Desired support was highest from doctors (59%) and psychologists (19%). A general request for support was associated with lower global health status (*p* = 0.03, odds ratio (OR) = 0.96, 95% CI: 0.92–0.99) according to EORTC QLQ-C30. Most of the assessed caregivers (*n* = 96) were life partners (64%; *n* = 61) who experienced higher distress than the corresponding patients (caregivers: 6.5 ± 2.5 vs. patients: 5.3 ± 2.4). When patients were on chemotherapy, caregivers indicated DT ≥ 6 significantly more frequently than patients themselves (*p* = 0.02).

**Conclusion:** Our data showed that glioma patients and their caregivers were both highly burdened. The PPQ allowed us to evaluate the psychosocial support requested and perceived by patients, detect supportive care needs, and provide information at a glance. Patients in poorer clinical condition are at risk of having unmet needs. The caregivers' burden and unmet needs are not congruent with the patients' need for support. In particular, caregivers of patients on chemotherapy were more highly burdened than patients themselves.

## Introduction

The prognosis in high-grade glioma patients remains poor, and supportive care needs should be addressed in a timely manner. The requirement for psychosocial support and early palliative has not only become a focus of (neuro-) oncological research (1–5), but is also incorporated in guidelines for the provision of care for these patients (6, 7). Application of patient-reported outcomes (PROs) has become essential in assessing the patients' quality of life and their needs, distress, and psychosocial burden, as well as supportive care needs (8). Recently, it has been shown that monitoring symptoms via PRO measures can be very helpful for cancer patients and even influence survival (9, 10).

Palliative and end-of-life care are still partially neglected topics in current neuro-oncological outpatient care. Seibl-Leven et al. reported recently that palliative care in glioblastoma patients is either not provided at all, or not in a timely fashion, leading to a shortage of services for patients and caregivers (11). Adequate assessment of unmet needs, early integration of palliative care and timely end-of-life-care planning should be implemented as clinical routine (12).

As glioma patients suffer from neurocognitive deficits caused by both the disease itself and the treatment (13–15) they may not always be able to answer PRO questionnaires. Furthermore, as reported by our group and others, patients undergoing chemotherapy or in a poor clinical condition may be missed by PRO assessment; however, at the same time, they are those who could particularly benefit from early supportive care (5, 16–18). Therefore, we believe it is of utmost importance to adequately identify glioma patients in need of supportive care.

Caregivers face challenging situations as well, sometimes even more so than glioma patients. The neurological, psychological and cognitive symptoms of patients with gliomas represent significant challenges to their caregivers: Not only do they have to cope with the diagnosis of the family member, with the therapy and the knowledge that they will finally lose their partner or parent or child, but also accept changes in roles, relationships, social isolation, financial restriction and sooner or later, have to take care of the partner or family member day and night (19–23).

Therefore, the aims of our study were to (1) Assess support received and needed by patients and (2) Assess support needed by patients' caregivers.

## Patients and methods

### Patients

During April 2015 to June 2016, patients at three German neuro-oncological centers were approached during their outpatient visits and asked to participate in the study. Inclusion criteria were diagnosis of glioma WHO grades II–IV regardless of disease stage (initial diagnosis or recurrent disease), absence of aphasia impairing communication or consent to the study, and given informed consent. Patients were asked to complete several PRO measures. Furthermore, demographic and clinical data were recorded in a database.

### PRO measures used

#### Distress thermometer (DT)

The DT is a self-reporting screening instrument developed by the National Comprehensive Cancer Network to evaluate psychological distress on a visual analog scale (0–10 points). A problem list with 40 items is included for patients to indicate the area of concern (family, financial, and physical) (24). Studies have proven its acceptance in oncological patients, and the German version for brain tumor patients was first evaluated by Goebel and Mehdorn (25). A score ≥6 indicates significant burden in brain tumor patients.

#### European organization for research and treatment of cancer quality of life questionnaire core module for cancer patients accompanied by the brain-specific module (EORTC QLQ-C30 + BN20)

The EORTC QLQ-C30 is a widely accepted questionnaire applying a 4-point Likert scale to evaluate cancer patients' quality of life. Five functional, three symptom, and six single-item scales as well as the global health status are investigated (physical, role, emotional, social, and cognitive functioning; fatigue, nausea and vomiting, pain; dyspnea, insomnia, appetite loss, constipation, diarrhea, and financial difficulties). Its validity and reliability have been proven in numerous clinical studies, and it is available in 85 languages. The additional module for brain tumor patients (BN20) consists of 20 questions specifically assessing their symptoms (3 neurological deficit scales, 1 future uncertainty scale, treatment and disease-related symptoms) (26, 27). The EORTC scores were calculated according to the user manual (28). Each scale is scored from 0 to 100, with higher scores indicating better functioning for functional scales and worse symptoms for symptom scales. In our regression and correlation analyses, we used the global health scale (GHS) as the primary endpoint.

#### The patients' perspective questionnaire (PPQ)

The “Patients' Perspective Questionnaire” (PPQ) is a questionnaire assessing patients' current status of support received, its subjective benefit and further needs. It was adapted for brain tumor patients based on a questionnaire used by Singer et al. (29). They applied several versions, whereas in our study we combined them into one questionnaire, added questions and items of probable interest to glioma patients according to the authors' experiences. This resulted in one questionnaire comprising three parts: Part I: The first 9 items assessed psychosocial distress (sad /worried /angry /tense /hopeful /burdened by disease/ burdened by other problems/ sufficiently supported/ sufficiently informed) by 5-step Likert-scales (scoring from 1 = not at all to 5 = very much) and if support was requested by the patient with regard to the respective item (“I need support for….”: yes/no). The latter questions were considered for the general request for support if one or more item was answered with “yes.” The next 10 items (Part II) assessed support provided and its subjective benefit on a 5-step Likert scale (scoring from 1 = support was not helpful at all to 5 = support was very helpful). The last 7 items (Part III) recorded support requested currently by the patients (from doctors, psychologist, social worker, and so on) with dichotomous answer possibilities (yes/no). Further support currently requested by any profession or next of kin was considered positive if one or more answers were “yes.” The questionnaire is provided as Supplement 1.

#### The questionnaire for the caregivers/caregivers' perspective questionnaire (CPQ)

In order to provide a questionnaire for glioma patients' caregivers in line with the PPQ with regard to structure and practicability, we combined elements and items after conducting a literature search and using an expert panel in the study group. We first applied it as a pilot study in family members volunteering during an information-sharing event for brain tumor patients. According to their anonymous feedback, the questionnaire was adapted with respect to wording, font size and item specification. The final version of the questionnaire is provided as Supplement 2.

Part I assessed the psychological distress on a visual analog scale (0–10 points) according to the DT applied to patients. Further, possible problem-items were provided similarly to the DT item list with dichotomous choices with regard to practical, family or emotional problems (24). In part II, we incorporated two questions with regard to quality of life and global health with Likert scales scoring from 1 to 7 according to the items 29 and 30 of the EORTC QLQ-C30 questionnaire (26). Finally, part III provided a list of items recording the support requested by caregivers (psychologists, social care, doctor, physiotherapy, dietician, self-help, friends, family members, palliative care). The questionnaire further included a question if an explanation of any term in the questionnaire was needed (Supplement 2).

#### Patients' and caregivers' assessment

Patients completed the DT, EORTC QLQ-C30+BN20 and the PPQ by themselves. Further, patients were asked directly by the attending neuro-oncologist during the patient-doctor consultation if they would like support by a psychologist. Neuro-oncologists also indicated and recorded their own assessment with regard to patients' unmet psycho-oncological needs independent of the assessment by questionnaires after having obtained the current medical history.

In a subgroup, patients' caregivers were assessed simultaneously by the CPQ developed for the study.

#### Statistical analysis

Demographic and tumor-related data, as well as Karnofsky-Index, were analyzed descriptively. Explorative Spearman's rho correlations between DT score as well as GHS and general request for support or request for support by doctors were performed.

Multivariate logistic regressions were performed with regard to “request for support in general” as well as “request for support by doctors” and “by other health care professionals.” Clinical and demographic factors probably influencing request for support were selected content driven by the authors as follows: sex (male/female), living situation (alone/in relationship), educational level (university degree/no university degree), WHO grade (low-grade/high-grade glioma), Karnofsky-Index (continuous variable, score 0–100), on chemotherapy (yes/no), GHS (continuous variable, EORTC QLQ-C30, score 0–100), DT score (continuous variable, score 0–10), surgery for recurrent disease (yes/no), and age (continuous variable, 25–85). The statistical analyses were performed using SPSS version 22 (IBM Corp., Armonk, NY).

#### Ethics

The study was performed in accordance with national law, institutional ethical standards, and the Declaration of Helsinki after approval of the study protocol by the local ethics committees (Mainz, Germany and Ulm/Günzburg, Germany [No: 837.349.15 (10117)]. All patients provided written informed consent prior to data assessment.

## Results

### Patients

Two hundred and thirty-two patients were assessed and 84% of the patients had a high-grade glioma. Mean age was 52 years (range 25–85). Male to female ratio was 1.25:1. Most of the patients were in a relationship and 30% (*n* = 71) had a higher education level. Mean Karnofsky-Index was 79 and 52% of the patients were on chemotherapy during assessment. Further details are provided in Table 1.

**Table 1:** Clinical and demographic data of the patient sample and results of the psychosocial assessment using the Distress Thermometer (DT; score ≥ 6 indicated significant burden in brain tumor patients), the European Organization for Research and Treatment of Cancer Quality of Life Questionnaire (EORTC QLQ)-C30+BN20, and the Patients' Perspective Questionnaire (PPQ).

**Variable**	**Patients *n* = 232 (100%)**
**AGE IN YEARS**	
Mean (SD; min, max)	52 (14; 20, 87)
**Sex**, n (%)	
Male	129 (56)
Female	103 (44)
**LIVING SITUATION**, ***n*** **(%)**	
Single	49 (21)
In relationship	75 (72)
Unknown	8 (7)
**EDUCATION LEVEL**	
University degree	71 (30)
No university degree	151 (66)
Unknown	10 (4)
**WHO-GRADE**, ***n*** **(%)**	
LGG (WHO I°+II°)	35 (16)
HGG (WHO III°+IV°)	190 (84)
**TUMOR LOCALIZATION I**, ***n*** **(%)**	
Frontal	99 (43)
Temporal	57 (25)
Parietal	31 (13)
Occipital	12 (4)
Other	17 (8)
Unknown	16 (7)
**ONGOING CHEMOTHERAPY**, ***n*** **(%)**	
Yes	111 (48)
No	121 (52)
**SURGERY FOR RECURRENT TUMOR**, ***n*** **(%)**	
Yes	57 (25)
No	140 (60)
Missing	35 (15)
**KARNOFSKY-INDEX**	
Mean (SD, range)	79 (16; 40–100)
**TIME SINCE DIAGNOSIS IN MONTHS**	
Mean (SD, range)	42 (54; 4–288)
Median	19
VALUE OF DISTRESS-THERMOMETER*	
Mean (*SD*, range)	5.0 (2.5; 0.0–10.0)
< 6 (*n*, %)	126 (54)
≥ 6 (*n*, %)	95 (41)
Missing	11 (5)
**SELECTED EORTC QLQ-C30 AND EORTC QLQ-BN20 SCORES, MEAN**	
(***SD***)	
C30 Global Health Status/QoL	57.4 (23.0)
C30 Physical functioning	68.8 (30.1)
C30 Role functioning	57.3 (36.4)
C30 Emotional functioning	60.4 (28.0)
C30 Cognitive functioning	58.3 (32.7)
C 30 Social functioning	54.3 (35.9)
C 30 Fatigue	46.3 (32.0)
C 30 Pain	23.4 (28.0)
BN20 Future uncertainty	43.9 (29.9)
BN20 Motor dysfunction	25.0 (27.1)
BN Communication	31.7 (32.4)
BN Headache	29.7 (32.2)

*LGG, low grade glioma; HGG, high grade glioma*.

### Patients' perceived and requested support according to the PPQ

We observed that 38% (87) of patients felt depressed (indicated ≥ 3 on the Likert scale), 44% (103) were anxious and 39% (91) were tense/nervous. Fifty-nine percent (138) reported to be adequately informed about the disease and therapy and 77% (180) of the patients felt sufficiently supported (Table 2).

**Table 2:** Results of assessments in 232 patients using the PPQ.

**Item**	Do you feel…? (Mean on Likert scale^*^)	**Support requested (*n*, %)**
**PART I: MOOD AND WELL-BEING**		
Sad/depressed	2.3 (1.2)	37 (16)
Worried/anxious	2.5 (1.2)	36 (16)
Angry	2.0 (1.2)	28 (12)
Tense/nervous	2.3 (1.2)	33 (14)
Hopeful	3.3 (1.2)	25 (11)
Burdened by disease	2.9 (1.3)	44 (19)
Burdened by other problems	2.2 (1.3)	27 (12)
Sufficiently supported	3.9 (1.3)	33 (14)
Sufficiently informed	3.5 (1.3)	49 (21)
**Profession**	**Received support (*****n*****, %)**	**If yes, how helpful? (Mean on Likert scale**^#^**)**
**PART II: RECEIVED SUPPORT**		
Doctor	157 (68)	3.9 (1.0)
Outpatient care/palliative services	46 (20)	3.8 (1.1)
Physiotherapist	54 (23)	3.6 (1.3)
Social service	27 (12)	3.4 (1.6)
Psychologist	31 (13)	3.3 (1.6)
Pastor	11 (5)	2.9 (1.7)
Dietician	12 (5)	2.8 (1.4)
Support group	4 (2)	2.3 (1.6)
Family	174 (75)	4.5 (0.9)
Friends	156 (67)	4.2 (1.0)
**Profession**	**Requested support (*****n*****, %)**
**PART III: REQUESTED SUPPORT**		
Doctor	137 (59)	
Outpatient care/palliative services	31 (13)	
Social service	26 (11)	
Psychologist	43 (19)	
Pastor	10 (4)	
Dietician	34 (15)	
Support group	23 (10)	

*In part I, patients indicated their psychological well-being and mood as well as satisfaction and unmet information needs. In part II, patients indicated received support and rated how helpful the support was. In part III, patients indicated by which profession he/she needed support*.

* *Likert scale 1–5 with 1 = not at all and 5 = very much*.

# *Likert scale 1–5 with 1 = not at all helpul and 5 = very helpful*.

Patients' support was reported to be highest from family (75%) and doctors (e.g., physician or attending neuro-oncologists, 68%). Only 13% were supported by psychologists. The support was mostly reported as helpful with highest mean scores being that of family and friends (mean score > 4), followed by doctors, outpatient care services and physiotherapists.

Desired support was highest from doctors (59%) and psychologists (19%) as well as from dieticians (15%, Table 2).

### Patients' requested support in comparison to DT, GHS, and clinical condition

Patients requesting support according to the PPQ generally showed higher DT scores (according to DT, *p* = 0.006, *r*_*s*_ = 0.20), lower GHS (according to EORTC QLQ-C30, *p* < 0.001, *r*_*s*_ = −0.34), and lower Karnofsky indices (*p* = 0.03, *r*_*s*_ = −0.18). The request for support was further associated with patients' wish for psychological intervention when asked directly (*p* = 0.03, *r*_*s*_ = 0.27), and the neuro-oncologist's clinical assessment of patients' unmet needs (*p* = 0.001, *r*_*s*_ = 0.24).

### Factors associated with current requested general support according to the PPQ

With regards to the request for support in general, we observed that patients reporting “lower GHS” were at higher risk for unmet needs (*p* = 0.03, odds ratio (OR) = 0.96, 95% CI: 0.92–0.99), as assessed by logistic regression analysis (step 5). Patients “living alone” indicated higher request for support; however, this was not significant. Educational level, WHO grade, Karnofsky-Index, on chemotherapy, DT score, surgery for recurrent disease and age were not significantly associated with the request for support in general.

### Factors associated with current requested support by doctors according to the PPQ

Logistic regression analyses revealed that “living situation/not in partnership” was associated with request for support by doctors according to the PPQ (living situation/in partnership *p* = 0.01, OR = 0.06, 95% CI: 0.01–0.53). Further, we observed a tendency of patients “on chemotherapy” to wish for greater support; however, this was not significant. Request for support from physicians was not significantly associated with educational level, GHS, sex, WHO grade, Karnofsky-Index, DT score, surgery for recurrent disease and age.

### Factors associated with current requested support by any health care profession according to the PPQ

With regard to request for support by any health care profession, logistic regression revealed that “living situation/not in partnership” as well as “university degree” were associated with wish for support by any health care profession according to the PPQ; “living situation/in partnership” was protective (*p* = 0.01, OR = 0.076, 95% CI: 0.10–0.57), whereas having “university degree” posed a higher risk (*p* = 0.04, OR = 7.86, 95% CI: 1.10–56.08). There was no significant association between requested support from any health care profession and sex, GHS, WHO grade, Karnofsky-Index, on chemotherapy, DT score, surgery for recurrent disease and age.

### The caregivers' burden

In a subgroup of 96 patients, their caregivers completed the CPQ. Patients' age in this subgroup was 56 years (*SD* 14.8, range 19–84). Most of the caregivers (64%, *n* = 61) were life partners.

Caregivers' DT mean was higher than the DT mean of the corresponding patients (caregivers: *DT* = 6.5, *SD* = 2.5 vs. patients: DT mean = 5.3, *SD* = 2.4). Similarly, a DT score ≥6 was reported more frequently by caregivers (55%, *n* = 53) than patients (47%, *n* = 37). Simultaneously, according to the CPQ, caregivers were highly burdened: 48% indicated to be anxious, 54% were sad and 70% reported concerns/worries. Practical problems were mostly problems with insurance or financial problems (24%), mobility and transport (38%) and working situation (25%). The changes in relationship (32%) and problems in interactions with spouse or life partner (28%) were frequently reported. Further results are presented in Table 3.

When patients were on chemotherapy, caregivers indicated DT ≥ 6 significantly more frequently than the patients themselves (patients: 33%, *n* = 13 vs. caregivers: 59%, *n* = 23, *p* = 0.02, Fishers' exact test).

**Table 3:** Results of assessments in 96 caregivers using the self-developed questionnaire.

**PART I: DISTRESS AND PROBLEMS**	
**Distress mean (SD; median)**	6.5 (2.5, 6)
**Problems with…**	*n* (%)
Child Care	11 (11)
Housing situation	10 (10)
Insurance/finance	23 (24)
Mobility/transport	36 (38)
Working/education	24 (25)
Interaction with children	10 (10)
Interaction with life partner	27 (28)
Interaction with parents	12 (13)
Depression	12 (13)
Anxiety	46 (48)
Nervousness	45 (47)
Sadness	52 (54)
Worries	67 (70)
Changes in relationship	31 (32)
**PART II: QUALITY OF LIFE**	
Quality of Life of caregiver^*^	4.4 (1.5)
Global health of caregiver^*^	4.7 (1.4)
**PART III: REQUESTED SUPPORT**	
**Profession**	*n* (%)
Psychologist	14 (15)
Social service	12 (13)
Doctor	23 (24)
Outpatient care	13 (14)
Physiotherapist	14 (15)
Pastor	4 (4)
Dietician	10 (10)
Self-help group	4 (4)
Family	25 (26)
Palliative services	8 (8)
Request for further explanation of terms	3 (3)

*In part I, caregivers indicated their distress and problems. In part II, caregivers indicated their quality of life and health status. In part III, they indicated by which profession he/she needed support*.

* *Likert scale 1–7 with 1 = extremely poor and 7 = very good*.

#### Caregivers' requested support

Twenty-eight percent (*n* = 26) of caregivers indicated a moderate and 14% (*n* = 13) a poor quality of life (mean of all assessments QoL: 4.4, and global health: 4.7 on a Likert scale 1–7). Requests for support came mostly from family (26%), doctors (24%), psychologists (15%), physiotherapists (15%) as well as social service (13%). Table 3 presents results in more detail.

## Discussion

In our study, we were able to apply the PPQ to glioma patients and assess support received and needed by patients as well as evaluate the accompanying caregivers by a study-specific questionnaire in a subgroup of patients. We found clinical and demographic factors (e.g., GHS, living situation, and university degree with regard to education) to be associated with higher wish for support, either global or support by a specific profession. Caregivers were even burdened more highly and needed support as well.

### Patients and required support

In our sample of glioma patients, we observed that the male to female ratio, general conditions expressed by the Karnofsky-Index and the high rate of high-grade gliomas (HGG) represented patients seen by neuro-oncologists in outpatient settings in general. However, as we did not assess the percentage of patients refusing the assessment, we are unable to reflect on the reasons for refusal (e.g., the assessment comprising three questionnaires might not have been well-accepted and probably too demanding). Seibl-Leven et al. reported a relatively high refusal and drop-out rate in their field study (11). Further, in a previous study by our group, we observed that patients refusing an assessment or dropping out of an observational study were more often with recurrent diseases, poorer clinical condition and harbored more often a glioblastoma (30, 18). Therefore, we should take into account that a certain percentage of patients probably with unmet needs may have been missed by our assessment and assume that we observed a selection of glioma patients, leading to a lower generalizability of the results.

In general, high burden and a strong wish for support for certain mental/emotional states (e.g., worries and depression) were indicated by the patients using the PPQ, which was also reflected by the fact that higher DT scores were associated with more frequent requests for support. This is in line with other studies and emphasizes how demanding comprehensive care for glioma patients is (2, 11, 31–34).

Interestingly, 15% of the patients required support by dieticians. While rarely addressed by neuro-oncologists, this aspect should be taken into account when planning supportive care for glioma patients as they frequently suffer from dysphagia in the final phase of the disease (35, 36).

Although many patients suffered from depression, worries and sadness, only 13% were supported by psychologists and 20% by outpatient/palliative care services, and only 19% and 13% required support from these respective professions. One reason for this finding could be that patients felt stigmatized when they experienced psychological problems and hesitated to ask for and accept support. Doctors rarely refer patients proactively for psychosocial support or palliative care timeously. Ideally, the treatment team is multidisciplinary from the very beginning of the disease trajectory. Even if the patients are not healed, this team can still support the patients together (5, 7, 36). This is also strengthened by the fact that according to the findings from the PPQ in our study, many patients and caregivers required general support as well as support from doctors relatively often. As we did not define the specialist disciplines (e.g., neurologist, family doctor or oncologist) in the questionnaire, it remains difficult to interpret this high percentage. It may probably include other disciplines: Patients might hesitate to indicate the need of psychological support; however, they might prefer to indicate support by doctors. The high rate of required support by doctors compared to other disciplines could be further due to the timing of the assessment. All patients were assessed prior to the appointment. Thus, potentially relevant questions were not addressed. Patients participating in this study may have attempted to be a “compliant patient” which may have introduced a certain bias in our study. This is also a possible explanation for the finding that patients rated the support by doctors as “helpful” in part II of the PPQ.

Patients seemed to be not well informed. This is of concern and needs to be considered seriously, and as already reported by other studies on patient-doctor consultations in (neuro-) oncology, improvement in communication skills is required (37). Glioma patients suffer from neurocognitive deficits and comprehension can be impaired (38, 39). Further, due to the lack of data and effective treatment options for patients with recurrent gliomas, patients may feel that they are under-informed and hope for new therapeutic options (40).

### Distress, GHS and request for clinical support

We found a correlation of elevated DT, higher burden of patients and request for clinical support. Furthermore, the wish of patients when asked directly and the assessment of attending neuro-oncologists were both associated with request for general support. It is well known that doctors' views are not always congruent with the patients' views with regard to psychosocial distress and unmet needs (2). However, our data show that if there is no time for an extensive psychosocial assessment or patients are unable to complete questionnaires during the routine clinical visit, a higher level of distress directly indicated by the patient on a visual analog scale (which should be feasible for most of the patients) may signal unmet needs. Of note, the doctor in charge has to find out during the consultation the reasons for the distress and the areas of unmet needs. In our opinion, a routinely implemented question on distress during the consultation could draw attention to the patients' problems and initiate support whenever another type of screening (e.g., with questionnaires) is not possible.

### Factors associated with required support

We observed several clinical and demographic factors to be associated with required support. As also shown by others, patients in poor general condition (as perceived by themselves and expressed in our study by GHS) require greater support (34, 41).

In times of increasing social isolation, it seems to be an important finding of our study that patients living alone were at risk of higher unmet needs. Attending neuro-oncologists and physicians should consider the social situation of patients in order to initiate support timeously, particularly as patients with gliomas (as well as their families) are at risk for social isolation *per se* (42, 43).

Interestingly, patients with higher educational level longed for more support by any health care profession than did other patients. Possibly, they may have been better informed than the others or needed further support to deal with information obtained by themselves (e.g., via the internet). Presumably, all patients deal intensively with the poor prognosis, the clinical deficits, the neurocognitive impairment and psychological burden; however, those with higher education verbalize their questions better than the others. Although our analyses have to be regarded as exploratory, we observed similar results as others and the factors could serve as features signalizing patients with unmet needs to the doctors in charge (22, 34).

### Caregivers' burden

As in other studies, the caregivers of our patients reported high distress, even higher than that of the patients (1, 2, 22, 23). Our caregivers were mostly life partners accompanying their relative to the consultation. It is well known that in glioma patients, family problems occur due to role changes, or changes in relationships. Demanding financial situations and practical issues also lead to tremendous burden in caregivers along the disease trajectory—reflected in our data as well (e.g., 70% of the caregivers reported to be worried). Hence, special screening for caregivers is required using instruments such as our questionnaire which was well accepted. When patients are on chemotherapy, neuro-oncologists should take into account that mostly caregivers organize the family life, take care of the patients and are in charge when patients suffer from side effects. In order to relieve caregivers, early integration of palliative care, outpatient services, social services and interdisciplinary treatment (during ongoing tumor-specific therapies) are required (44–47).

### Caregivers' requested support

Although caregivers reported high burden, only a minority wished for support by any profession. This may be partially due to functional coping strategies [e.g., high expectation of self-efficiency (20)] as well as feelings such as shame and fear about being unable to manage. These hinder caregivers to requesting and/or accepting “external help.” Some patients may also refuse outpatient care services as they do not like to accept the help of people they do not know and wish to stay at home in the final phase as well (36). This could lead to an enormous burden for caregivers when not supported by outpatient palliative care, which is the task of the doctor in charge (mostly physicians and neuro-oncologists) to initiate in a timely and sensitive way (45).

### Strengths and limitations of the study

Of note, this is the first study that applied the two questionnaires in glioma patients and caregivers simultaneously in a multicenter study, with both questionnaires found to be useful for clinical and research purposes. However, the study does have several limitations.

The study required patients to be fit enough to complete several questionnaires. Therefore, patients in advanced stages of the disease and with cognitive deficits were not included, even though they might be the ones with the highest level of distress and need for supportive care. Shorter assessments are required for such patients. The study was a cross-sectional study and did not investigate the course of needs and distress during the disease trajectory. PPQ and CPQ were not validated instruments; therefore, the results should be interpreted with caution. Furthermore, the patient population was in a heterogeneous disease stage and current treatment. However, this can also represent a certain benefit as the results translate well to the general outpatient population seen at neuro-oncological centers. The content-driven selection of probable influencing clinical and demographic factors with regard to requested support for explorative correlation and logistic regression analyses reduced the statistical strength.

## Conclusion

Our data show that glioma patients are highly burdened, and doctors play a crucial role in initiating these patients' psychosocial care and support. The PPQ allows us to evaluate the support requested and perceived by the patients, to detect supportive care needs and provide information at a glance. We observed clinical factors (e.g., when patients live alone in relation to support by doctors, lower GHS with regard to general requested support) and demographic factors (e.g., living alone or higher educational level with regard to support by any profession) that are possibly associated with unmet needs.

Of note, the patients' needs do not always reflect the caregivers' situations. Especially, caregivers of patients on chemotherapy are more burdened than patients themselves. Therefore, either using a questionnaire or questioning during consultation, regular assessment of relatives/caregivers accompanying the patient is required.

## Author contributions

MR contributed to the conception and design of the study and wrote the first draft of the manuscript. DM, HL, EW, and MD assessed the study data and organized the data base. MR and DM developed the questionnaire for caregivers. CW, FR, and SS contributed to the conception and design of the study and edited the manuscript. JC contributed to the conception and design of the study, performed the statistical analyses, and edited the manuscript. All authors contributed to revision of the manuscript, read and approved the submitted version.

### Conflict of interest statement

The authors declare that the research was conducted in the absence of any commercial or financial relationships that could be construed as a potential conflict of interest.
