# Supplementary material for: Supportive Care Needs in Glioma Patients and Their Caregivers in Clinical Practice: Results of a Multicenter Cross-Sectional Study
Source: Front Neurol. 2018 Sep 11;9:763. doi: 10.3389/fneur.2018.00763 (PMC6141995; doi:10.3389/fneur.2018.00763)
Supplement: Supplementary file 1 [file Table_1.DOCX]

**Patients‘ perspective questionnaire**

Many patients are burdened by the disease. Some problems are described in this questionnaire and we would like to know if you wish support for any problem. Please take some time to fill in our questionnaire spontaneously.

Participation is on a voluntary basis. Do you have any questions? Please don't hesitate to ask a member of our study team.

**Part I, please indicate how you feel and if you are affected by any of the listed emotions.**

| **During the last week I felt…** | **Not at all………….very much** | **I wish support for this emotion**  **Yes No** |
| --- | --- | --- |
| 01 …sad/depressed | 1 2 3 4 5 | □ □ |
| 02 …worried/anxious | 1 2 3 4 5 | □ □ |
| 03 …angry | 1 2 3 4 5 | □ □ |
| 04 …tense/nervous | 1 2 3 4 5 | □ □ |
| 05 …hopeful | 1 2 3 4 5 | □ □ |
| 06 …burdened by the disease (e.g. diagnosis, prognosis, therapy side effects, pain and other) | 1 2 3 4 5 | □ □ |
| 07 …burdened by other problems (e.g. working situation, family problems, financial problems and other) | 1 2 3 4 5 | □ □ |
| 08… sufficiently supported (by family, friends, doctors or others) | 1 2 3 4 5 | □ □ |
| 09…sufficiently informed about the disease (e.g. therapy options, clinical studies and others) | 1 2 3 4 5 | □ □ |

**Part II, please indicate, if you received support by any of the listed group of persons and if yes, how helpful the support was.**

| Since the last visit in our outpatient clinic….  **Did you receive support by….** | Yes No | **If yes, how helpful was the support?**  **Not at all…. Very helpful** |
| --- | --- | --- |
| 01 Doctor | □ □ | 1 2 3 4 5 |
| 02 Mobile care/palliative services | □ □ | 1 2 3 4 5 |
| 03 Physiotherapist | □ □ | 1 2 3 4 5 |
| 04 Social service | □ □ | 1 2 3 4 5 |
| 05 Psycho-oncologist | □ □ | 1 2 3 4 5 |
| 06 Pastor | □ □ | 1 2 3 4 5 |
| 07 Dietician | □ □ | 1 2 3 4 5 |
| 08 Support group | □ □ | 1 2 3 4 5 |
| 09 Family | □ □ | 1 2 3 4 5 |
| 10 Friends | □ □ | 1 2 3 4 5 |

**Part III, please indicate, if you wish support by any of the listed group of persons.**

| **Do you need support by….** | Yes No |
| --- | --- |
| 01 Doctor | □ □ |
| 02 Mobile care/palliative services | □ □ |
| 03 Social service | □ □ |
| 04 Psycho-oncologist | □ □ |
| 05 Pastor | □ □ |
| 06 Dietician | □ □ |
| 07 Support Group | □ □ |

**Thank you for your participation!**
