# Supplementary material for: Supportive Care Needs in Glioma Patients and Their Caregivers in Clinical Practice: Results of a Multicenter Cross-Sectional Study
Source: Front Neurol. 2018 Sep 11;9:763. doi: 10.3389/fneur.2018.00763 (PMC6141995; doi:10.3389/fneur.2018.00763)
Supplement: Supplementary file 2 [file Table_2.DOC]

**Caregivers’ perspective questionnaire**

Dear caregivers,

we would like to interview you in order to find out which unmet needs for support you have regarding the psycho-social distress you feel and your quality of life.

Participation is on a voluntary basis.

Do you have any questions? Please don't hesitate to ask a member of our study team.

**Distress**

Please mark this scale from 0 to 10 with a cross, depending on the level of distress you feel:


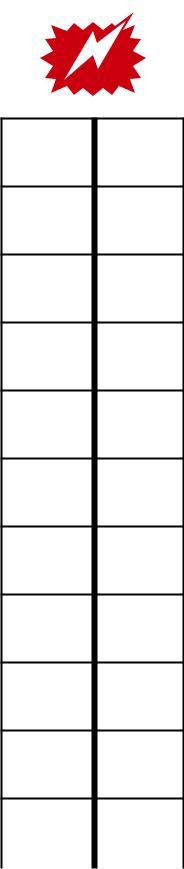


| **highest distress** | **10** |
| --- | --- |

**9**

**8**

**7**

**6**

**5**

**4**

**3**

**2**

**1**

| **no distress** | **0** |
| --- | --- |

**1**

Please mark if you have problems in any of these topics. Please make sure you *answer every question* with yes *or* no!

**Did any of the listed problems concern you due to the illness of your relative?**

**YES** **NO** **PROBLEM**

childcare

living situation

insurance/finance

mobility/transportation

work/education

**family interactions**

with children

with partner

with parents

**emotional problems**

depression

anxiety

nervousness

sadness

worries

changes in your relationship

**Quality of life**

Please answer the following questions by marking one of the numbers from 1 to 7 with a cross, depending on the situation you are currently experiencing:

1. How would you estimate **your own quality of life** during the last week?

**very poor** ➀ ➁ ➂ ➃ ➄ ➅ ➆ **very good**

2. How would you estimate **your own health status** during the last week?

**very poor** ➀ ➁ ➂ ➃ ➄ ➅ ➆ **very good**

**2**

Would you like to receive support **for yourself** by:

**Yes** **No**

**Psycho-oncologist**

**Social Service**

**Doctor**

**Mobile care**

**Physiotherapist**

**Spiritual support; Pastor**

**Dietician**

**Self-help group**

**Relatives**

**Friends**

**Palliative care**

Do you need any further explanation with regard to topics or the above mentioned professions or do you have any questions?

yes   
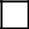
 no


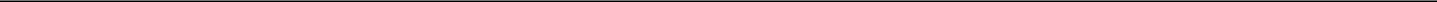

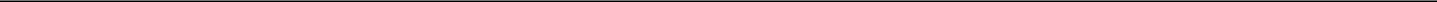

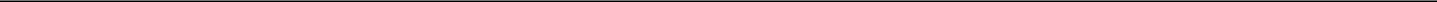


How are you related to the patient?

| spouse/ life partner | brother/sister | friend |
| --- | --- | --- |


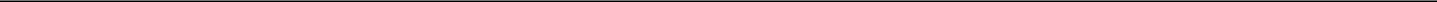

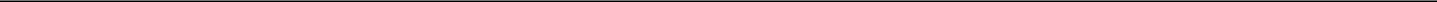

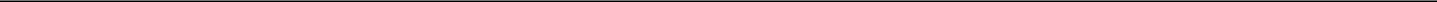


**Thank you for your participation!**

**3**
